# Toward reliable population estimates of wolves by combining spatial capture-recapture models and non-invasive DNA monitoring

**DOI:** 10.1038/s41598-018-20675-9

**Published:** 2018-02-01

**Authors:** J. V. López-Bao, R. Godinho, C. Pacheco, F. J. Lema, E. García, L. Llaneza, V. Palacios, J. Jiménez

**Affiliations:** 1 Research Unit of Biodiversity (UO/CSIC/PA), Oviedo University, 33600 Mieres, Spain; 2 CIBIO/InBio-Centro de Investigação em Biodiversidade e Recursos Genéticos, Universidade do Porto, 4485-661 Vairão, Portugal; 3 Fuentes Amarelle 6, 15130 Corcubión, Spain; 4 A.RE.NA, Asesores en Recursos Naturales, S.L, 27003 Lugo, Spain; 5 Instituto de Investigación en Recursos Cinegéticos-(CSIC-UCLM-JCCM), 13071 Ciudad Real, Spain

## Abstract

Decision-makers in wildlife policy require reliable population size estimates to justify interventions, to build acceptance and support in their decisions and, ultimately, to build trust in managing authorities. Traditional capture-recapture approaches present two main shortcomings, namely, the uncertainty in defining the effective sampling area, and the spatially-induced heterogeneity in encounter probabilities. These limitations are overcome using spatially explicit capture-recapture approaches (SCR). Using wolves as case study, and non-invasive DNA monitoring (faeces), we implemented a SCR with a Poisson observation model in a single survey to estimate wolf density and population size, and identify the locations of individual activity centres, in NW Iberia over 4,378 km^2^. During the breeding period, posterior mean wolf density was 2.55 wolves/100 km^2^ (95%BCI = 1.87–3.51), and the posterior mean population size was 111.6 ± 18.8 wolves (95%BCI = 81.8–153.6). From simulation studies, addressing different scenarios of non-independence and spatial aggregation of individuals, we only found a slight underestimation in population size estimates, supporting the reliability of SCR for social species. The strategy used here (DNA monitoring combined with SCR) may be a cost-effective way to generate reliable population estimates for large carnivores at regional scales, especially for endangered species or populations under game management.

## Introduction

Estimating the abundance of species is one of the most contentious issues in conservation and applied ecology^1,2^. Decision-makers in wildlife policy require reliable population size and density estimates to adopt and justify interventions. Reliability is essential to build acceptance and support in management decisions and, ultimately, trust in managing authorities. Otherwise, speculation and distrust can emerge after decisions are made, and may undermine entire management or conservation strategies^1,3^. Incorrect population estimates may lead to misinterpretations of the status of populations, the impact of interventions (e.g., hunting quotas or culling programs), or the degree to which conservation goals have been achieved.

The management of large carnivores is controversial due to the multiple political, socio-economic and conservation interests involved. Information on population size or the impact of interventions is in constant demand, not only by managers, researchers and conservationists, but also by other interest groups. This is exemplified by recurrent debates around large carnivore numbers, particularly centred on endangered and charismatic species, such as in the case of tigers (*Panthera tigris*), lions (*Panthera leo*) or wolves (*Canis lupus*)^4–8^. Clear population targets are often established by managing authorities, and have become political issues, with reliable assessments of changes in large carnivore ranges and population size required to justify actions^9^.

Wolves are a good example of a species whose estimates of population size and range are systematically demanded by multiple actors (e.g., governments, livestock producers, conservationists), and whose population targets are often established, compared to other wildlife. Wolf estimates are required to meet legal obligations in both Europe^10^ and the US^11^. The Spanish authorities approved a short-term recovery goal of 15 packs for the endangered Sierra Morena wolf population^12^. The Swedish parliament decided to maintain its wolf population within a minimum of 20 reproductions and a maximum of 210 individuals^13^. The Norwegian parliament has established a national target of four to six wolf litters a year^14^. The recovery goal for wolves in the Northern Rocky Mountains after reintroduction into Yellowstone National Park and central Idaho, US, was set to >300 wolves and >30 breeding pairs evenly distributed among the recovery areas for three consecutive years^15^.

The number of wolf packs or reproductions are often the basis of wolf monitoring^16–18^, and may make wolf population size estimates more homogeneous in a transboundary and regional context. Notwithstanding, many wolf management strategies still combine the number of packs/reproductions with the number of individuals. In Europe, for example, estimates reporting the number of individuals still prevail^19^, requiring a conversion exercise from the number of packs/reproductions (usually using estimates on pack size). However, this conversion remains a challenge^20^ and, in most cases, the data required to calculate conversion factors properly is not available. While the number of packs or reproductions are a reasonable target for wolf monitoring at regional scales^17^, apart from mismatches between management goals (i.e., often based on the number of individuals, for instance, to establish hunting quotas) and monitoring targets (i.e., packs or reproductions), there are cases where estimating the number of wolves may be important. Examples include small and endangered wolf populations, such as the Sierra Morena^12^ or Mexican^21^ wolf populations, and populations under game management^6^.

Different field methods and analytical approaches have been used to address the challenge of surveying large carnivore populations at regional scales^17,22–26^, including non-invasive DNA monitoring^21,23,27^. This method, combined with traditional capture-recapture procedures, is often presented as a promising strategy to achieve robust, feasible and economically affordable population size estimates. Nevertheless, several constraints have been identified when implementing this monitoring strategy. A recurrent limitation is the imprecision when it comes to defining the effective sampling area^28^. When using traditional capture-recapture procedures, additional spatial information is required in order to define the effective sampling area, such as information from collared individuals^29,30^. However, gathering this information may not be budgetary feasible in many cases, particular at regional scales. Moreover, the minimum number and type of collared individuals needed to capture the movement parameters in a population must be representative. On the other hand, encountering probabilities are heterogeneous among those individuals exposed to sampling^31^.

However, the development of spatially explicit capture-recapture approaches (SCR)–linking population size with space by estimating a latent variable representing the location and number of individual’s activity centres–allows the estimation of density, defined as the local intensity of a spatial point process^32^, taking into account heterogeneity in encounter probabilities. In SCR approaches, the effective sampling area is estimated based on the underlying spatial point process, using information from individual’s encounters across detectors^31^. Although it is possible to integrate other sources of spatial information, such as telemetry data^33^. For large carnivores, within the SCR framework, several field sampling methods have been used to generate spatial encounter data, mainly camera-trapping data^34–36^ and non-invasive DNA monitoring^37–39^. Occasions (i.e., repeated opportunities for observation) can be accomplished either in space or time (e.g., one site sampled multiple times or multiple sites sampled once). Under this framework, SCR approaches based on a single survey and using a Poisson observation model allows density estimates^40,41^.

SCR assumes that distributions of animal activity centres are uniformly and independently distributed over the state space (*S*) –the area that includes all individuals potentially exposed to sampling^32^. However, both assumptions are violated by multiple species. For example, in social and territorial carnivores, like wolves or lions, a large segment of the population lives in packs or prides, respectively. In solitary carnivores, like many felid species or bears^42^, adult females and juveniles (i.e., family groups) stay together for several months within the annual cycle before dispersion. In wolves, the functional unit is the pack, but wolf populations show a varying proportion of non-resident individuals^43^. Although the size of wolf home ranges is influenced by individual attributes (e.g., age, sex, reproductive status)^43,44^, pack cohesion varies throughout the annual cycle^45,46^ and pack members may use space differently within pack territories^47^, spatial overlap of home ranges for pack members being high^44,47^.

Here, we combined non-invasive DNA monitoring (faeces) with a SCR Poisson observation model in a single survey to demonstrate the performance of this monitoring strategy in estimating wolf density and population size, and identify the activity centres of wolves at regional scales. Using wolves as illustrative example, we used simulations to show how SCR models perform with species violating the assumptions that distributions of animal activity centres and animal movements are independent^32^. In addition, to provide additional support to this monitoring strategy, we compared estimates of the spatial scale parameter sigma (*σ*) -related with the movement of individuals- obtained from the SCR model, with empirical data from a set of collared wolves in the study area.

## Materials and Methods

### Sample collection and genetic analyses

**Figure 1:**
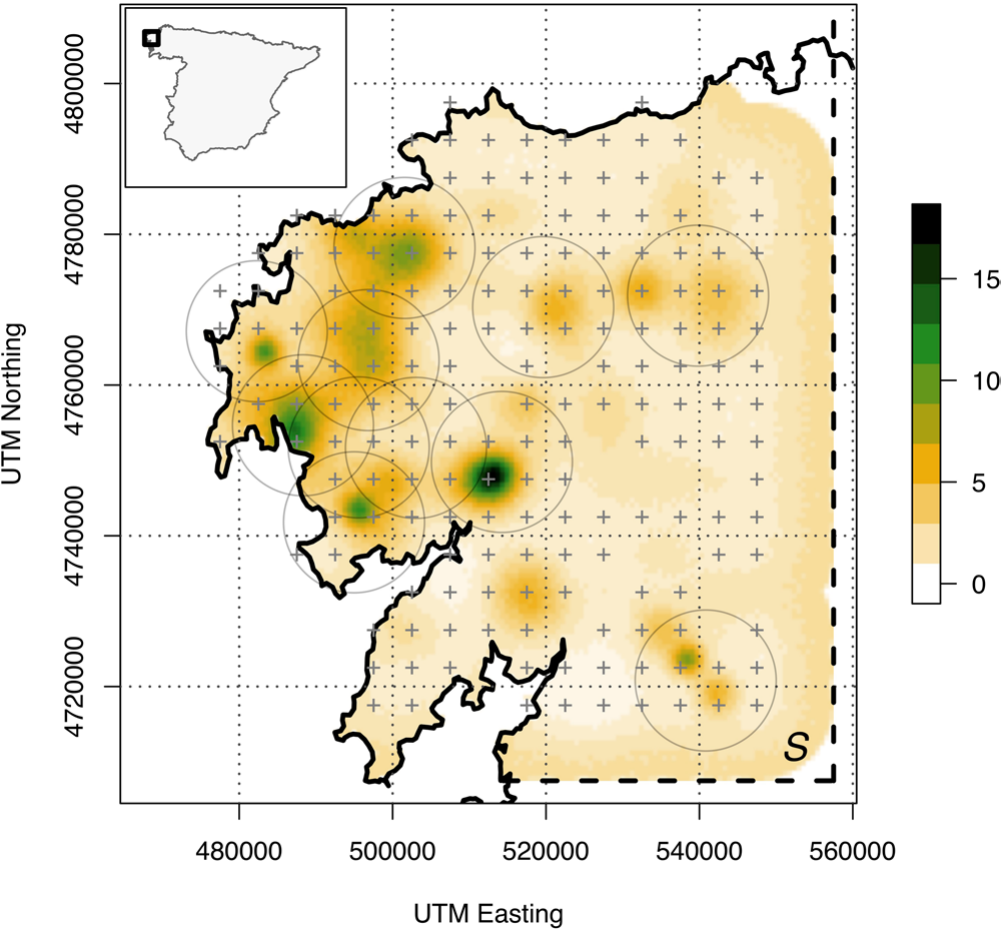
Posterior mean density of activity centres for wolves in the study area. The spatial locations of detectors are denoted by “+” (grey). *S*: state space. We included the approximate location of reproductive pack territories (grey circles) by creating a conservative buffer area centred on the rendezvous sites of known breeding packs in 2013^48^. The selection of the size of the circles was based on previous information on the mean and SD of wolf home range sizes of subadult/adult wolves belonging to packs in NW Iberia (122.1 ± 93.6 km^2^)^52^ (the area was equal to the mean plus SD = 215 km^2^). The figure was produced by José Jiménez using R^55^.

We tested the use of non-invasive DNA monitoring (faeces) with a SCR Poisson observation model in a single survey in the wolf population of Costa da Morte and surroundings (hereafter CM; Galicia, NW Spain) (Figs 1 and S4), a segment of the NW Iberian wolf population^18^ covering ca. 4,378 km^2^, and where 11 breeding packs were detected in 2013^48^. During the 2013 wolf breeding period (May-October, including the pup-rearing period), 317 wolf-like faeces were collected across CM. In collaboration with rangers from the Regional Government of Galicia, faeces were searched along transects distant from human settlements^26^. Random sampling is not effective to locate wolf faeces; therefore, scat sampling was focused on landscape elements often used by wolves as marking places (paths and trails, particularly focusing on junctions and mountain passes)^26^. A minimum of 10 km of transect length was invested per each 10 × 10 km UTM cell, surveying a total of ca. 750 km over the study area (Figs S1 and S2). Surveying a minimum of 10 km per cell resulted in a probability of detecting wolves of >0.6^17^, which is likely influenced by the persistence of wolf faeces. All samples were georeferenced using a GPS and preserved in 96% ethanol at room temperature.

Details on genotyping are provided in Pacheco *et al*.^49^ and Appendix S1. MtDNA species identification was achieved for 288 samples (91%). Based on DNA quality, 172 samples were further considered for individual identification using four replicas of 18 Ancestral Informative Markers^50^. After a Bayesian analysis to exclude dogs (see details in Pacheco *et al*.^49^), ninety-five wolf samples achieved a consensus genotype (55%) with <20% missing data, following rules defined in Godinho *et al*.^50^. Multiple factors can influence the success rate of genotyping and individual identification, such as the age of the wolf scat (related to the presence of odour)^51^. Nevertheless, the success rate observed in this study was similar to other values reported in studies using non-invasive DNA^51^. Estimated allelic dropout across loci was ADO = 0.033, and estimated false alleles were FA = 0.005^49^. After regrouping of identical genotypes^49^, we identified 65 individual wolves. This dataset had a probability of identity (PID) = 4.19 × 10^−8^ and a PIDsiblings = 4.01 × 10^−4^ (Appendix S1).

### Wolf estimates

We applied a SCR modelling approach to estimate wolf density, population size and the distribution of individual’s activity centres in CM. Conceptually, activity centres are points *s*_*i*_ representing “*where the animal i of the N population size lives*”. Formally, the sequence of points *s*_1_, *…*, *s*_*N*_ is a realization of a latent spatial point process, and allows the estimation of density using SCR models. SCR assumes that every individual *i* in the population has its own activity centre *s*_*i*_, and that all activity centres are distributed across the study area. Moreover, encounter probability (*p*_*ij*_) is a decreasing function of distance between the activity centre of the individual *i* (*s*_*i*_) and the location of the detector *j* (*x*_*j*_). Therefore, SCR addresses the movement of individuals by assuming that each individual has an activity centre, and that the probability of capture individuals is a function of the distance from the activity centres to detector locations. In our case, because the collection of wolf faeces was not attached to physical traps, our detectors were the centroids of the cells within the sample grid, and the effort invested in searching samples inside cells was used as a “detector level” covariate.

We built a grid cell layer with the size of cells being small compared to typical wolf home ranges. Considering previous information on the spatial ecology of wolves in NW Iberia, we created a 5 × 5 km (25 km^2^) cell grid layer over the study area. Cell size was well below the average (±SD) home range size for subadult/adult wolves belonging to packs in NW Iberia (122.1 ± 93.6 km^2^)^52^. This cell size was selected to avoid an excessive loss of resolution in the scale parameter sigma (*σ*) –a parameter that determines the decline of detection frequency of individuals in detectors with increasing distance from their activity centres–, resulting in 174 5 × 5 km cells in CM (Fig. S2). Each cell centroid was considered as a detector location (Fig. S1). Thus, we modelled centroids (detector locations) as count detectors because the same wolf can be detected at multiple cells during the sampling, and more than one individual can be detected in the same detector^32^. All genotyped faeces were assigned to its detector. The encounter histories are a bi-dimensional matrix *y* (*i* × *j*), because we used a single occasion.

Therefore, the number of times that an individual *i* is located in a detector *j* is Poisson distributed with mean λ_ij_:

1 $${\rm{y}} \sim {\rm{Poisson}}({{\rm{\lambda }}}_{{\rm{ij}}})\,$$

The link function between the location of detectors and the activity centres follows a half-normal distribution^32^:

2 $${{\rm{\lambda }}}_{{\rm{ij}}}={{\rm{\lambda }}}_{{\rm{o}}}\exp (-\frac{{d}_{ij}^{2}}{2{\sigma }^{2}})$$

where *d*_*ij*_ is the distance between the activity centre for each individual *s*_*i*_ and the detector location *x*_*j*,_ and *λ*_0_ is the baseline encounter probability. The baseline encounter probability is modelled with a log function being dependent on sampling effort within each cell, and *σ* is the Gaussian scale parameter for the distance function between activity centres and detector locations (Fig. S2):

3 $$\mathrm{log}({{\rm{\lambda }}}_{0}[j])={\alpha }_{0}+{\alpha }_{1}\times {\rm{L}}[j]$$

As several cells share land and sea surfaces in CM (Fig. 1), we used the density of transects (km km^−2^) in each terrestrial section of the cells as a covariate accounting for sampling effort ${\rm{L}}[j]$. Density of transects was scaled and centred, and *α*_0_ and *α*_1_ were the parameters to be estimated. The total number of activity centres (*N*) were calculated using the data augmentation approach^32^. All-zero encounter histories (500 potential individuals) were added to encounter histories of detected individuals. We generated the state space (*S*) adding a 10-km buffer to the detector grid. This distance must be >2.5 × *σ*^32^. The resulting *S* was ca. 7,500 km^2^. We used an application of the “zero’s trick” to buffer an irregular detector array (i.e., near the coastline) without discretizing the state space^53^. We scaled and centred *S* dividing the spatial coordinates (m) by 10,000^32^.

We fitted a null Poisson distributed observation model (*M*_0_) in a Bayesian framework using Nimble v.0.6-2^54^ and R^55^ (Appendix S2). We ran 3 chains of the MCMC sampler with 50,000 iterations each, yielding 150,000 total samples from the joint posterior distributions. To check for chain convergence, we assessed MCMC convergence and mixing by visually inspecting trace plots for each parameter of interest, and we calculated the Gelman-Rubin statistic *R-hat*^56^ using the R package *coda*^57^, where values below 1.1 indicated convergence. We evaluated the goodness of fit of the model by using the Bayesian p-value approach and two fit statistics (described in Royle *et al*.)^32^: *i*) individual encounter frequencies, which evaluates heterogeneity in encounter frequencies due to space (i.e., in SCR models, the explicit factor space explains part of the heterogeneity); and *ii*) detector frequencies, which is based on aggregating over individuals and replicates to form centroid-encounter frequencies (Appendix S2).

SCR approaches assume that all individuals are uniformly and independently distributed over the state space *S*. We explored the influence of such violations on population density estimates by carrying out three simulation studies (described in Appendix S3). We tested for the influence of the randomness in the location of wolf packs across the state space, because the identification of the spatial location of activity centres is more accurate inside the detector grid.

### Spatial information on wolves

We evaluated the performance of this modelling approach by comparing *σ* from *M*_0_ with two sigma parameter estimates based on wolf spatial behaviour (${\hat{\sigma }}_{hr}\,{\rm{and}}\,{\hat{\sigma }}_{hr2})$. We used spatial information from 18 wolves collared in the study area between 2006 and 2014. Wolves were captured with Belisle^©^ leg-hold snares (Edouard Belisle, Canada), chemically immobilised by intramuscular injection of medetomidine (Domitor^®^, Merial, France; 0.10 mg/kg) and equipped with GPS-GSM collars (Followit, Sweden). Our dataset was comprised of 9 males and 9 females, of ≥2 years, and considering individuals integrated and non-integrated within packs. We used a total dataset of 5,432 locations (2 locations per day and wolf; mean number of locations per Wolf = 302, range 34–789). The average monitoring period for collared wolves was 161 days (range 14–397). In order to provide additional support to this modelling approach, we also compared sigma estimates during the same period of the year (${\hat{\sigma }}_{hr2})$, to account for variations in wolf spatial behaviour along the annual cycle^43^. To do this, we subset our dataset on wolf spatial information considering only those locations between March and October, corresponding with the period of DNA monitoring plus two previous months given by the potential persistence of wolf faeces in the field. This dataset was composed by 3,650 locations from 16 wolves (range 26–549 locations per individual). We estimated individual home ranges (km^2^) using the 95% fixed-kernel method with the R package *adehabitatHR*^58^.

We calculated the scale parameters ${\hat{\sigma }}_{hr}$ (considering all wolf locations) and ${\hat{\sigma }}_{hr2}$ (only considering those wolf locations overlapping with the DNA monitoring period) considering the average spatial requirements of wolves in CM, as follows:

4 $${\hat{\sigma }}_{hr}\text{or}\,{\hat{\sigma }}_{hr2}=\sqrt{\frac{A/{\rm{\pi }}}{{{\rm{q}}}_{2,{\rm{\alpha }}}}}$$

where ${{\rm{q}}}_{2,{\rm{\alpha }}}$ was the value of a Chi-square with 2 degrees of freedom (*α* = 0.05, ${{\rm{q}}}_{2,{\rm{\alpha }}}=5.99$) and *A* was the average home range area (m^2^)^32^.

We also evaluated the spatial mismatch between the spatially explicit posterior mean density of activity centres for wolves across *S* and the location of known breeding packs in CM. We used the information on the number and spatial location of wolf breeding packs from the official wolf monitoring survey carried out in CM in 2013^48^ (wolf monitoring was based on the location of breeding packs following Llaneza, García & López-Bao 2014)^26^.

Finally, we estimated the proportion of wolves that were part of known breeding packs. To do this, we assumed that in late summer, breeding wolf packs oscillated between 7 and 9 individuals, including the year’s pups. Although pack size is variable in wolves^43^, our assumption was based on 88 field observations of breeding packs at *rendezvous sites* (i.e., sites used by pack members and pups approximately in the first 5 months of pups’ age)^59^ in N Iberia, where the average number of observed pups was 4.82 (95%CI 4.57–5.08), the average number of subadults was 1.67 (95%CI 1.25–2.08) and the average number of adults was 1.98 (95%CI 1.74–2.22)^60^. Considering the number of known breeding packs in CM in 2013 (11 packs)^48^ and the range of individuals per pack in late summer (7–9 wolves), we transformed the number of packs into number of wolves belonging to packs.

#### Ethics statement

Wolves were captured under permits 19/2006, 71/2009, 86/2011, 095/2013 and 8765/2015 from the Regional Government of Galicia. All field procedures were carried out in accordance with animal welfare regulations.

## Results

From DNA analyses, forty-nine wolves (75%) were detected once, nine wolves were detected twice and, respectively, three, one and three individuals were detected 3, 4 and 5 times (the average number of captures by wolf was 1.46). The number of individuals captured per detector is shown in Table S1. Based on raw data (65 individual genotypes and 95 captures), asymptotic rarefaction curves were not reached (Fig. S3). All parameters from the null model (*M*_0_) showed convergence and R-hat < 1.1. The posterior density estimate $(\hat{D})$ for wolves in CM was 2.55 wolves/100 km^2^ (95% Bayesian Credible Interval (BCI) = 1.87–3.51; Table 1; Fig. S4). The posterior mean abundance of wolves, $\hat{N}$ (±SD) was 111.6 ± 18.8 individuals (95%BCI = 81.8–153.6). The posterior estimate for sigma was 0.33 (m, scaled by 10^4^) (95%BCI = 0.27–0.40; Table 1). Bayesian p-values showed a good fit for the case of individual encounter frequencies (p-value = 0.457), but not for detector-encounter frequencies (p-value = 0.000) (Fig. S5).

**Table 1:** Posterior summaries of parameters estimated from the SCR Poisson encounter model (null model, *M*_*0*_) to estimate wolf density.

	Mean	SD	BCI		
			2.5%	50%	97.5%
Density ($\hat{{\rm{D}}}$)	2.55	0.42	1.87	2.50	3.51
alpha1 (${\hat{{\rm{\alpha }}}}_{0}$)	−1.21	0.25	−1.38	−1.21	−0.74
alpha2 (${\hat{{\rm{\alpha }}}}_{2}$)	0.24	0.13	−0.01	0.24	0.50
psi (ψ)	0.38	0.07	0.27	0.38	0.53
sigma ($\hat{{\rm{\sigma }}}$)	0.33	0.03	0.27	0.32	0.40

Estimates were based on 3 chains of 50,000 iterations, and thin rate = 1, yielding 150,000 total samples from the joint posterior. BCI = Bayesian Credible Intervals. alpha1 (${\hat{{\rm{\alpha }}}}_{0}$) and alpha2 (${\hat{{\rm{\alpha }}}}_{2}$) are used to model the baseline encounter rate, being dependent on sampling effort (see main text for details): $\mathrm{log}({{\rm{\lambda }}}_{0}[j])={\alpha }_{0}+{\alpha }_{1}\times {\rm{L}}[j]$, where *L*_*j*_ is the density of transect in each cell; psi (ψ) is a parameter of the augmented data, and sigma (σ), or movement parameter, is the half-normal scale parameter that describes the rate at which detection probability declines as a function of distance.

From simulation studies, using clusters of individuals and repulsion between clusters (packs in our case) we tested the potential role of spatial aggregation and non-independence of individuals, and we only found a slight underestimation in population size estimates (Appendix S3). The adequacy of the Root-Mean-Squared-Error for the posterior mean for the parameter $\,\hat{N}$, and the coverage of 95% Highest Posterior Density intervals performed better when all clusters of individuals were located within the area covered by the detector grid (Appendix S3). Estimations of $\hat{N}$ slightly decreased in accuracy and precision when clusters of individuals where totally or partially outside the detector grid (Appendix S3).

The average total home range estimate for our set of collared wolves was 266.4 km^2^; whereas this figure was 233.1 km^2^ when we considered only locations within the interval March-October. The estimates for ${\hat{{\rm{\sigma }}}}_{hr}$ and ${\hat{{\rm{\sigma }}}}_{hr2}$ were very similar, 0.37 and 0.35, respectively (m, scaled by 10^4^). These values were within the 95% BCI of the posterior estimate for sigma from *M*_0_ (0.27–0.40; Table 1), indicating a good biological performance of the model. The posterior mean density of activity centres across the state space spatially matched all the approximate locations of detected breeding packs in CM in 2013 (Fig. 1). However, we also detected some activity centres that were not overlapping with known breeding packs, indicating potential pack locations. Finally, the estimated number of wolves not linked to packs was 23.6 ± 8.7 individuals (i.e., 16–25% of the total number of wolves estimated using SCR; 88.0 ± 16.7 wolves were estimated to belong to packs).

## Discussion

Controversy often surrounds population size and density estimates of large carnivores. Different pitfalls are usually highlighted, such as the lack of standardisation and homogenisation in sampling protocols, lack of replicability or high uncertainties in estimates. For example, the majority of population size estimates for large carnivores in Europe lack rough calculations of uncertainty^18,19^. There is a demand for development of reliable monitoring tools, including those that allow for the quantification of uncertainty. In this regard, several powerful approaches, paying attention to accuracy, precision and optimization, have been proposed^17,34,61,62^. Improving the reliability of population estimates is expected to increase the credibility of surveys and improve trust in the agencies and stakeholders in charge of large carnivore monitoring.

We combined non-invasive DNA monitoring (faeces) and a SCR approach, with a Poisson observation model using a single survey, to estimate density, population size and the location of activity centres in wolves at a regional scale. The number of sampling surveys can constrain the application of SCR approaches at regional scales. We used the null model *M*_0_ to estimate the number of wolves with a measure of uncertainty (111.6 ± 18.8) across an area of >4,000 km^2^. A critical point in SCR models is the need for sufficient spatial recaptures for reliable estimates of σ^32^. Considering the average number of captures by wolf (1.46), the number of individuals captured across detectors (65), and the space among detectors (5 km), our spatial parameters can be considered reliable^31,32^. Count-based observation models, such as the Poisson model, using multiple detections of the same individual at the same detector, and being based on a single survey, allows the estimation of model parameters successfully. For situations in which behavioural or time-related covariates –across the sampling occasions- are not available, the property of additivity of the Poisson distribution allows the aggregation of the observation data, facilitating the use of the Poisson model based on a single survey to estimate model parameters (the observation model is the same for all values of *K*)^32^. Nevertheless, when implementing encounter frequency models, like a Poisson model, encounter frequencies over short periods are mainly due to individual behaviour. Moreover, it can be questionable to consider that all encounters are independent, which deserves further investigation.

The location of wolf activity centres fully matched the approximate locations of detected breeding packs in CM. But we also detected non-overlapping aggregations of activity centres (Fig. 1), probably corresponding to other pack locations where no reproduction was detected in 2013, or where the criteria used to assign wolf reproduction were not satisfied^26,48^. In fact, the spatial location of some aggregations of activity centres corresponded with the location of previously known breeding packs in the study area^50^. The combination of both wolf surveys (packs and individuals), together with information on pack size^60^, allowed us to approximate the number of wolves not linked to breeding packs in late summer, between 16 and 25% of wolves in our case. This estimate is similar to those obtained during winter wolf monitoring in other study areas across the wolf range. The percent of non-resident wolves in winter monitoring in different North American wolf populations ranged from 7 to 20%^43^. Interestingly, the above comparisons suggest that when using the number of packs as a target for wolf monitoring, underestimation in population estimates may occur.

For species with large spatial requirements, such as large carnivores, and short study periods, the area of activity centres used during the sampling period may represent only a section of the total animals’ home range. In our case, the sampling period spanned several months during the breeding and pup-rearing periods, and the posterior estimate for sigma ($\hat{{\rm{\sigma }}}=0.33$) was close to the sigma calculation using the total home range from our dataset of collared wolves (${\hat{{\rm{\sigma }}}}_{hr}=0.37$), as well as the restricted dataset of locations for the period March-October (${\hat{{\rm{\sigma }}}}_{hr2}=0.35$) (note that both estimates were within the 95%BCI for sigma from *M*_0_; Table 1). Thus, using the SCR Poisson approach, we were able to capture some aspects of the spatial behaviour of wolves. Differences in scent marking patterns among individuals^26,27^ may be behind the small differences observed in sigma, which could lead to some small bias in population estimates. Young and lone wolves more often deposit their faeces off-trail compared to adult and territorial individuals^27,63^. On the other hand, the persistence of wolf faeces may facilitate the detection of scats and the occurrence of spatial recaptures.

The assumptions that all individuals are uniformly and independently distributed over the state space *S*^32^ are violated by multiple species, which raises concerns when using SCR methodologies^64^, and may also have prevented their use in some cases. In this study, our simulations show that SCR can be reliable in species violating these assumptions. The aggrupation of wolves in packs, and their territorial behaviour, had a minimum impact on population size estimates. We found a slight underestimation in population size estimates (Appendix S3)^32,65^. Importantly, our results highlight the importance of setting the detector grid appropriately when monitoring species in which individuals are aggregated. Outside the detector grid, the individuals from a cluster (i.e., pack), and randomly located, were detected fewer times and with fewer spatial and non-spatial captures (Appendix S3), resulting in less accuracy and precision for $\hat{{\rm{N}}}$ and σ. Therefore, we recommend that the detector grid be extended to cover all previous suspected/known clusters of individuals in a given study area, and the use of simulations to detect possible bias in estimates (Appendix S3).

The combination of non-invasive DNA monitoring (faeces, hair, etc.) and SCR modelling approaches can be used to monitor species at regional scales accurately, such as large carnivores and large herbivores. Reliable population size estimates are particularly important for these species considering that ca. 60% of the world´s largest carnivores and herbivores are classified as threatened with extinction on the International Union for the Conservation of Nature (IUCN) Red List^66^ and different populations are under game management. The use of a single survey is expected to be less costly compared to multiple visits to detectors^64^. Moreover, the use of this monitoring strategy is expected to have positive implications for the adaptive management of these species. Adaptive management is a systematic approach to improve resource management by learning from management outcomes^67^. Because it is a learning-based process^68^, monitoring is an essential step in the adaptive management process. In our case, the ability to obtain reliable population estimates – quantifying uncertainty - that are reproducible over time is fundamental to assess the impact of interventions properly, which can be achieved with this monitoring strategy.

Apart from funding to carry out genetic analyses, the main challenge in implementing this monitoring strategy is an appropriate sample collection to ensure a sufficient number of recaptures in a single survey. In this regard, an alternative to optimize the use of limited resources, without compromising the collection of samples, may be the integration of multiple trained stakeholders and volunteers in sample collection (see an example for brown bears, *Ursus arctos*, in Europe)^69^, which should be properly designed to record all required information, including sampling effort.

## Electronic supplementary material

Supplementary Information
