# Supplementary material for: Toward reliable population estimates of wolves by combining spatial capture-recapture models and non-invasive DNA monitoring
Source: Sci Rep. 2018 Feb 1;8:2177. doi: 10.1038/s41598-018-20675-9 (PMC5794931; doi:10.1038/s41598-018-20675-9)
Supplement: Supplementary file 1 — Supplementary Information [file 41598_2018_20675_MOESM1_ESM.docx]

Supplementary Information

Title: **Toward reliable population estimates of wolves by combining spatial capture-recapture models and non-invasive DNA monitoring**

López-Bao JV, Godinho R, Pacheco C, Lema FJ, García E, Llaneza L, Palacios V, Jiménez J

**Fig. S1.** **Map of the study area (Costa da Morte, Galicia, Spain) and centroids of the 10x10 km cells sampled (blue crossed, referred to as detectors in main text).** The size of the grey circles represents the number of captures (i.e., number of genotyped wolf faeces) at a detector. The map is displayed in Universal Transverse Mercator (UTM) Zone 29N north coordinates. The figure was produced by José Jiménez using R (R Core Team 2017).


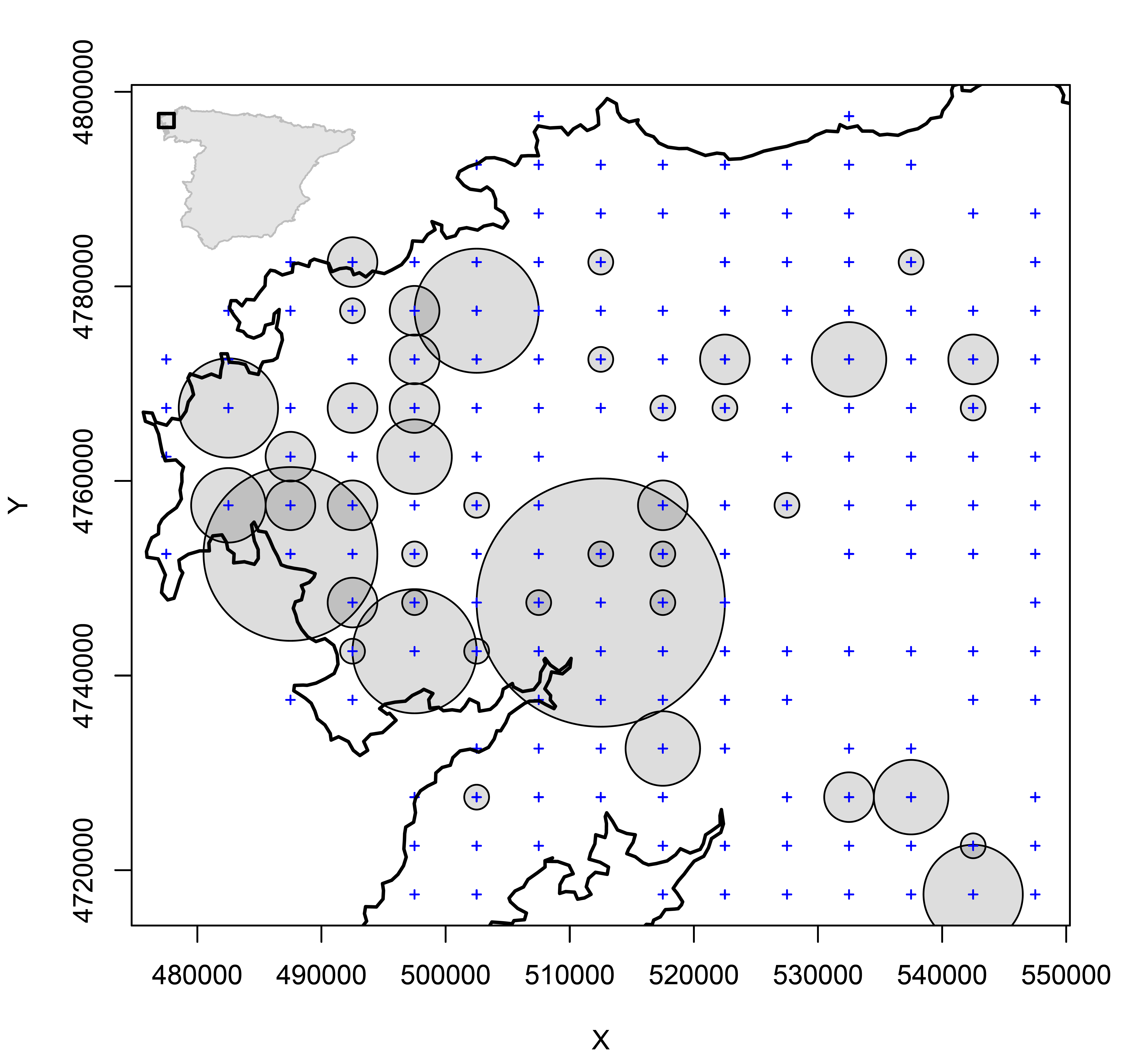


**References**

R Core Team (2017). R: A language and environment for statistical computing. R Foundation for Statistical Computing, Vienna, Austria. https://www.R-project.org/.

**Fig. S2. Sampling effort by cell.** We used as a covariate accounting for search effort ($L\left[ j \right]$ in main text), the density of transect length (km km^-2^) in each terrestrial section of the cells. The figure was produced by José Jiménez using R (R Core Team 2017).


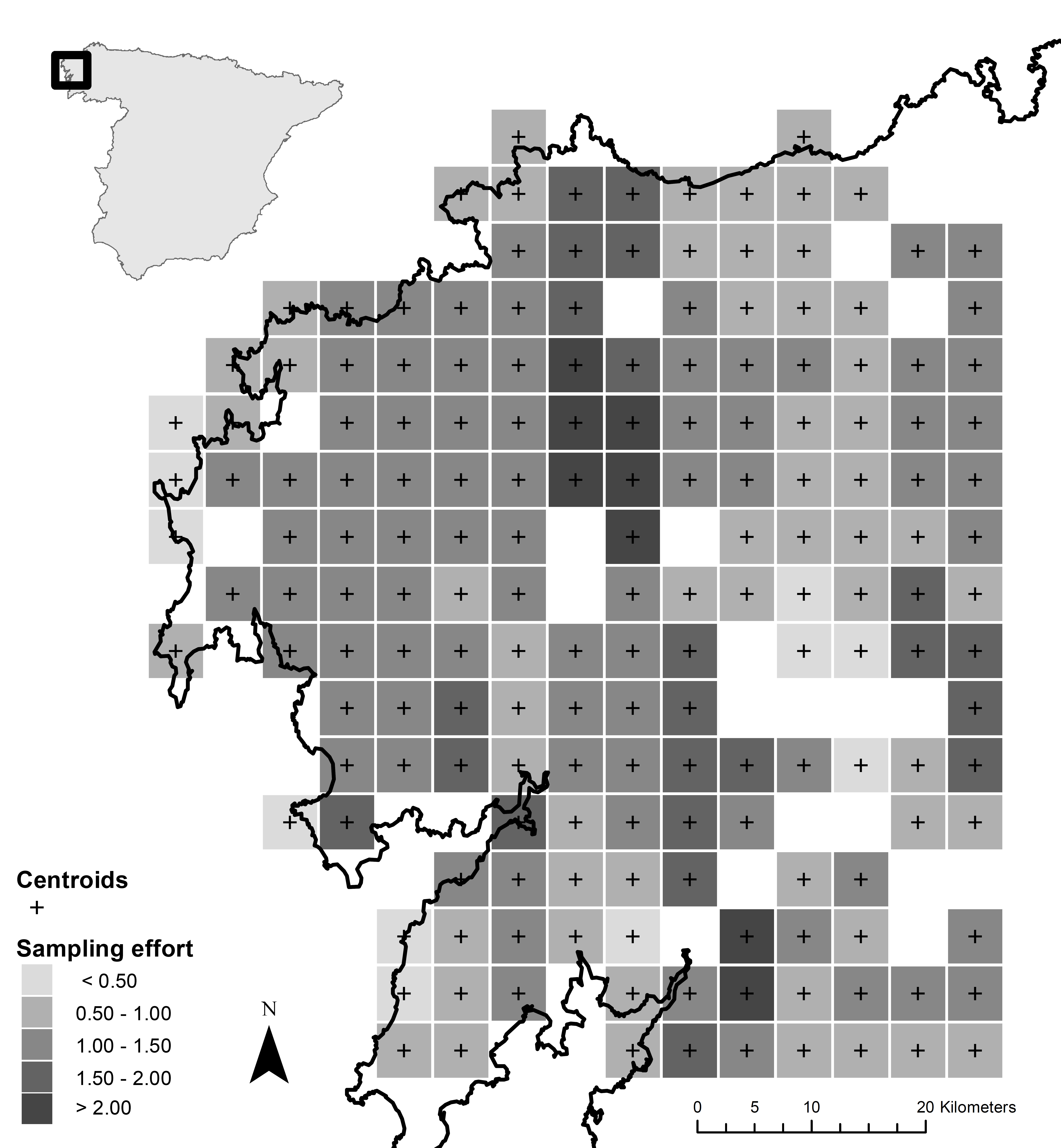


**References**

R Core Team (2017). R: A language and environment for statistical computing. R Foundation for Statistical Computing, Vienna, Austria. https://www.R-project.org/.

**Fig. S3. Rarefaction Curves for the 65 individual genotypes and 95 captures of our dataset.** We used three rarefaction curve methods by fitting the cumulative number of unique genotypes to the number of genotypes analysed using three equations (see details in: Kohn *et al.* 1999; Eggert *et al.* 2003, and the equation proposed by D.  Chessel in Valière 2002) using the script files provided by GIMLET 1.3.3. (Valière 2002) and the function “*nls2*” for R to fit our data to each equation. Input file data were randomised 1,000 times to avoid any influence in the estimation of parameters (Kohn *et al.* 1999). For each case, the mean of the asymptotes over the 1,000 curves was taken as the estimate of the number of individuals (Figure). Observed: circles, mean observed: black line, Kohn´s equation: red line, Chessel´s equation: green line, Eggert´s equation: blue line. The figure was produced by Raquel Godinho using GIMLET 1.3.3. (Valière 2002) and R (R Core Team 2017).


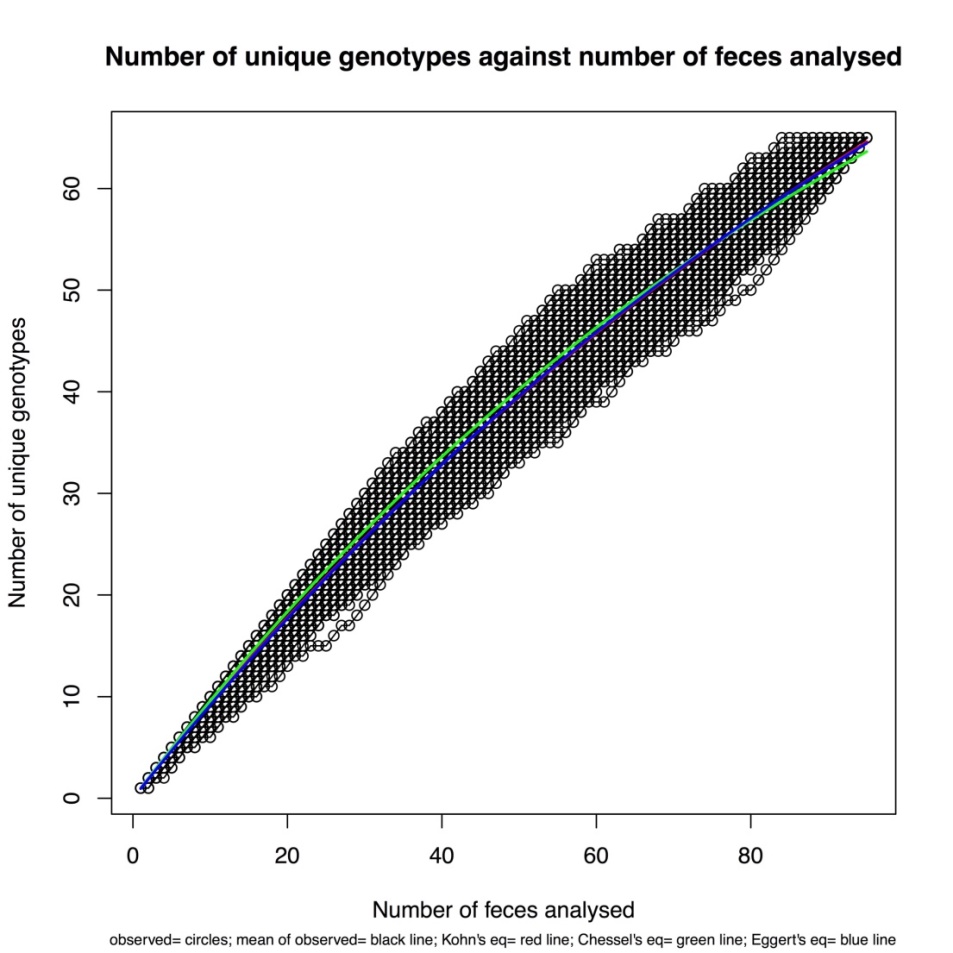


Note that for the 65 individual genotypes and 95 captures of our dataset rarefaction curves were not asymptotic. The asymptote for Kohn´s equation was estimated at 240.7±100.9 individuals. The asymptote for Eggert´s equation was estimated at 137.4±50.4 individuals. The asymptote for Chessel´s equation was estimated at 109.8±9.4 individuals.

**References**

Eggert, L.S., Eggert, J.A., & Woodruff, D.S. (2003). Estimating population sizes for elusive animals: the forest elephants of Kakum National Park, Ghana. *Molecular Ecology*, **12**, 1389-1402.

Kohn, M.H., York, E.C., Kamradt, D.A., Haught, G., Sauvajot, R.M., & Wayne, R.K. (1999). Estimating population size by genotyping faeces. Proceedings of the Royal Society London, **266**, 657-663.

R Core Team (2017). R: A language and environment for statistical computing. R Foundation for Statistical Computing, Vienna, Austria. https://www.R-project.org/.

Valière, N. (2002). GIMLET: a computer program for analysing genetic individual identification data. *Molecular Ecology Notes*, **2**, 377–379.

**Fig. S4. Bayesian posterior density distribution for estimates of the density of Iberian wolves** **in Costa da Morte, Galicia, Spain.** Dotted line denotes the mode from the 150,000 total samples from the joint posterior distribution of density (Density ($\hat{D}$) in Table 1, main text). The figure was produced by José Vicente López-Bao using R (R Core Team 2017).


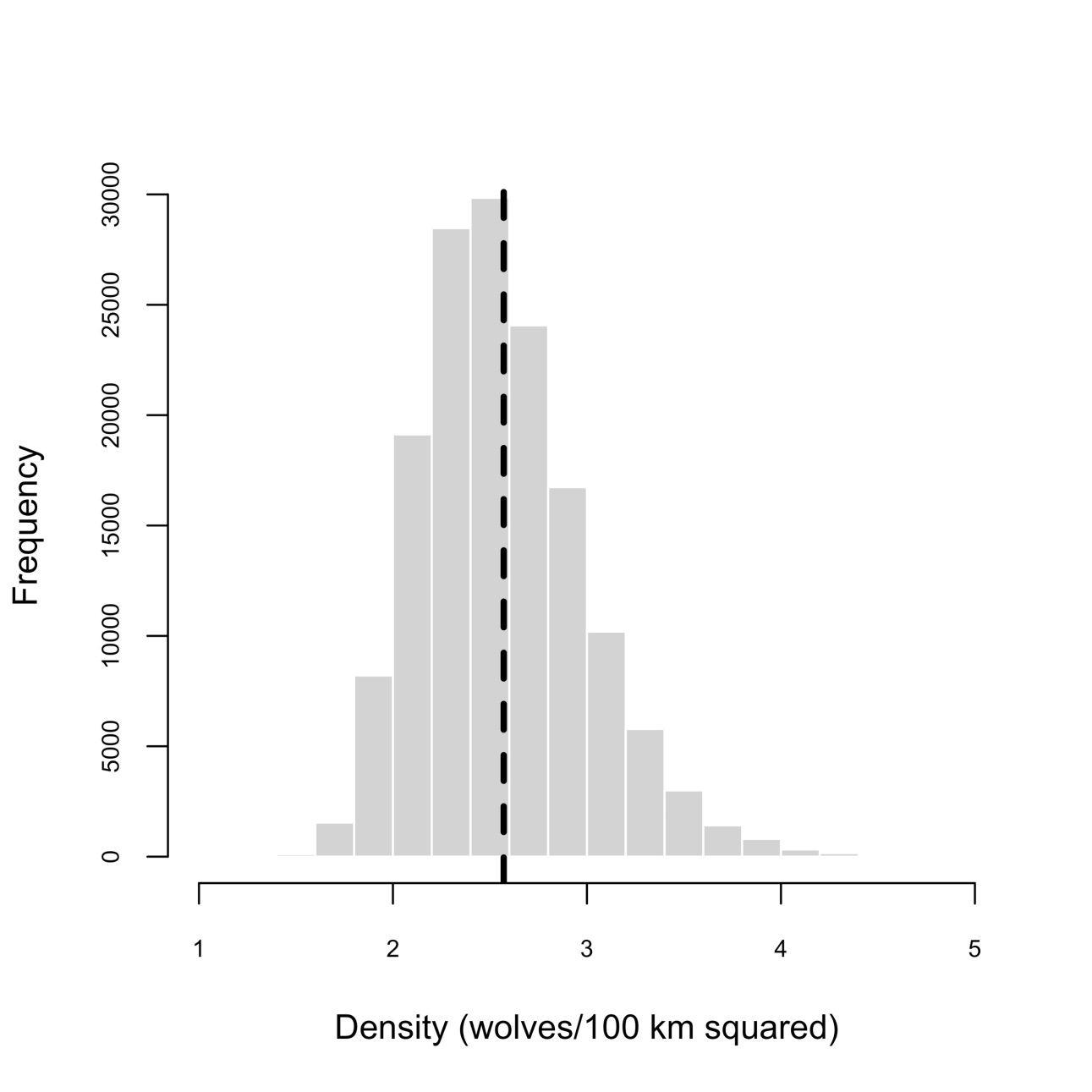


**References**

R Core Team (2017). R: A language and environment for statistical computing. R Foundation for Statistical Computing, Vienna, Austria. https://www.R-project.org/.

**Fig. S5. Scatter plot of replicate versus actual discrepancy measures for SCR-Poisson model *M_0_*.** The Bayesian p-values are the proportion of points above the 1:1 equality line. Bayesian p-values showed a good fit for the case of individual encounter frequencies (p-value = 0.457), but not for trap-encounter frequencies (p-value = 0.000).


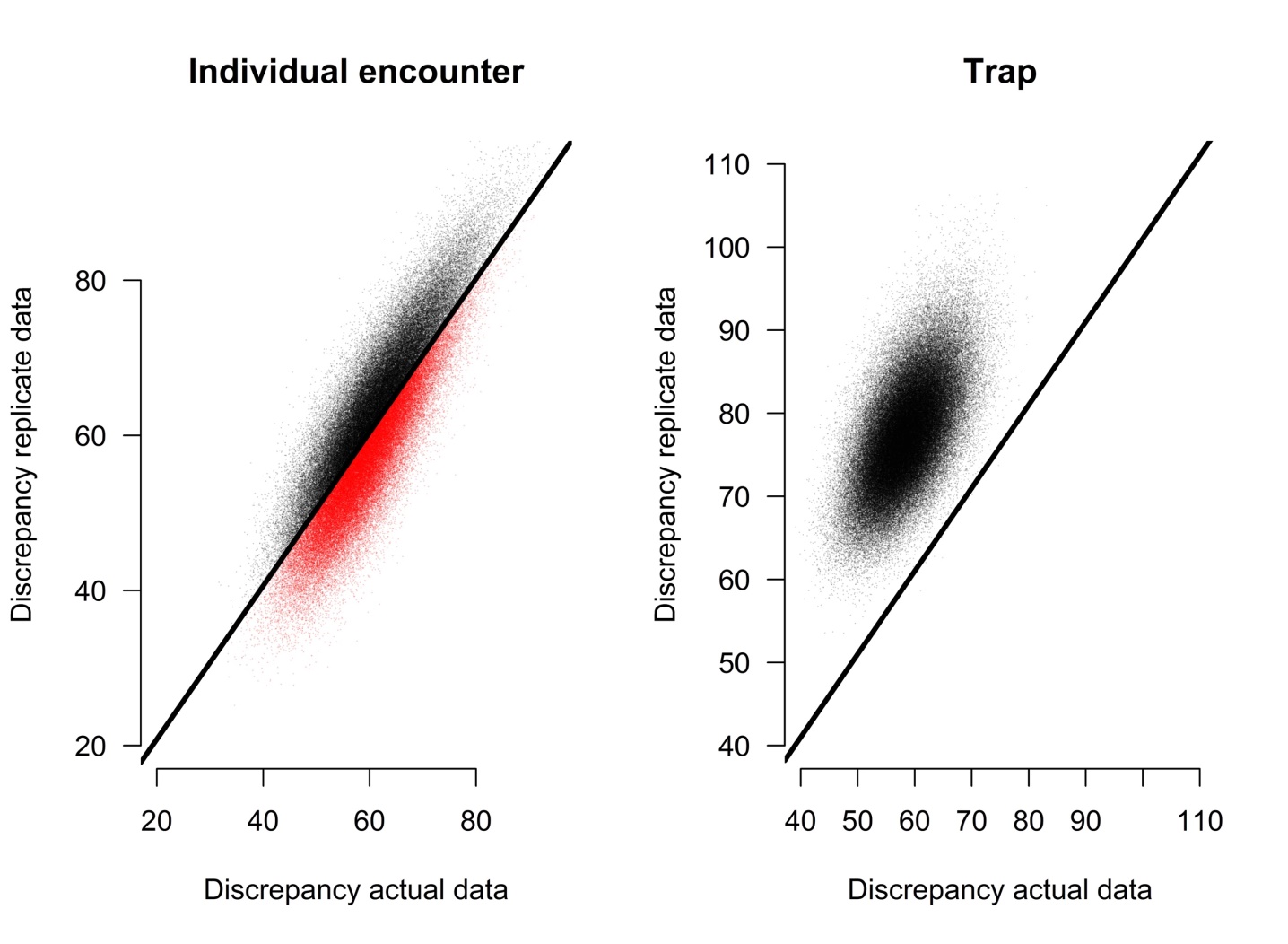


**References**

R Core Team (2017). R: A language and environment for statistical computing. R Foundation for Statistical Computing, Vienna, Austria. https://www.R-project.org/.

**Table S1. Distribution of the number of individuals captured per detector.**

| **Number of individuals** | **Number of detectors (%)** |
| --- | --- |
| 0 | 131 (75%) |
| 1 | 19 (11%) |
| 2 | 13 (7.5%) |
| 3 | 5 (3%) |
| 4 | 2 (1%) |
| 5 | 2 (1%) |
| 6 | - |
| 7 | 1 (0.5%) |
| 8 | - |
| 9 | - |
| 10 | 1 (0.5%) |

**Appendix S1.** **Details on genotyping of wolf faeces.**

Extraction and manipulation of DNA from scats was confined to dedicated rooms with sterile conditions and positive air pressure. DNA extraction followed Frantz *et al.* (2003) after the GuSCN/silica procedure (Boom *et al.* 1990). Potential PCR inhibitors were further removed using pre-rinsed Microcon® YM-30 centrifugal Filter Units (Millipore, Billerica, MA). Negative controls were included throughout the process to monitor for potential DNA contamination. We used the mitochondrial DNA control region (Vilà *et al.* 1999) for species identification. Successful amplifications were sequenced using BigDye chemistry (Applied Biosystems). Sequencing products were analysed on an ABI3130xl genetic analyser and aligned using SEQSCAPE 2.5 (Applied Biosystems). Individual identification was assessed based on 18 Ancestral Informative Markers (AIMs) (Godinho *et al.* 2015), specifically selected for Iberian wolves (based on F_ST_ values between wolves and dogs, 15 markers, and probability of identity, 3 markers). The set of markers included 17 microsatellites and a 5 bp deletion at intron 3 of the KIT-ligand gene (KITLG) (Pacheco *et al.* 2017). DNA quality to proceed to genotyping was assessed following Godinho *et al.* (2015). AIMs were amplified in two-steps using a pre-amplification protocol (Smith et al., 2011) and markers were amplified in four multiplex reactions using the Multiplex PCR Kit (QIAGEN) in a 10 μl final volume. Four replicas of each PCR step were performed. For the first PCR, we used approximately 5 ng of DNA and a concentration of 0.2 μM of unlabelled primers. On the second PCR, forward primers were M13-tailed to follow a fluorescent labelling protocol (Blacket *et al.* 2002). Details on thermocycling conditions are provided in Pacheco *et al.* (2017). PCR products were separated by size on an ABI3130xl genetic analyser. Alleles were scored against the GeneScan500 LIZ size standard, using GENEMAPPER 4.0 (Applied Biosystems) and manually checked twice independently.

Two initial replicas of each genotype were used to perform a maximum likelihood estimate of genotyping errors (allelic dropout and false alleles) using PEDANT 1.0 (Johnson & Haydon 2007). These estimates were then used to determine the minimum number of replicas needed to minimize genotyping errors using GEMINI 1.3.0 (Valière & Berthier 2002). This number was set to four. Consensus genotypes over four replicas were assembled following rules defined in Godinho *et al.* (2015). Samples with more than 20% missing data were removed from the analysis. Identical genotypes were filtered using GIMLET 1.3.3 (Valière 2002) and discarded. The parameter probability of identity (PID) and probability of identity for siblings (PIDsiblings) were estimated using GIMLET 1.3.3 (Valière 2002).

Bayesian clustering analysis, implemented in Structure 2.3.4 (Pritchard *et al.* 2000), was used to identify wolves, dogs and possible hybrids by assessing individual membership proportions (*qi*) and their 90% Bayesian Credible Intervals (BCI) to two inferred clusters (K=2) (see details in Pacheco *et al*. 2017). Only wolves were considered for further analyses.

**References**

Blacket, M.J., Robin, C., Good, R.T., Lee, S.F., & Miller, A.D. (2002). Universal primers for fluorescent labelling of PCR fragments - an efficient and cost-effective approach to genotyping by fluorescence. *Molecular Ecology Resources*, **12**, 456–463.

Boom, R.C.J.A., Sol, C.J., Salimans, M.M., Jansen, C.L., Wertheim-van Dillen, P.M., & Van der Noordaa, J.P.M.E*.* (1990). Rapid and simple method for purification of nucleic acids. *Journal of Clinical Microbiology*, **28**, 495–503.

Frantz, A.C., Pope, L.C., Carpenter, P.J., Roper, T.J., Wilson, G.J., Delahay, R.J., & Burke, T. (2003). Reliable microsatellite genotyping of the Eurasian badger (*Meles meles*) using faecal DNA. *Molecular Ecology*, **12**, 1649–1661.

Godinho, R., López‐Bao, J.V., Castro, D., Llaneza, L., Lopes, S., Silva, P., & Ferrand, N. (2015). Real‐time assessment of hybridization between wolves and dogs: combining noninvasive samples with ancestry informative markers. *Molecular Ecology Resources*, **15**, 317-328.

Johnson, P.C.D., & Haydon, D.T. (2007). Software for quantifying and simulating microsatellite genotyping error. Bioinform. Biol. *Insights* **1**, 71.

Pacheco, C., López-Bao, J.V., García, E., Lema, F.J., Llaneza, L., Palacios, V., Godinho, R. (2017). Spatial assessment of wolf-dog hybridization in a single breeding period. *Scientific Reports*, **7**, 42475.

Pritchard, J.K., Stephens, M., & Donnelly, P. (2000). Inference of population structure using multilocus genotype data. *Genetics*, **155**, 945–959.

Smith, M.J., Pascal, C.E., Grauvogel, Z.A.C., Habicht, C., Seeb, J.E., & Seeb, L.W. (2011). Multiplex preamplification PCR and microsatellite validation enables accurate single nucleotide polymorphism genotyping of historical fish scales. *Molecular Ecology Resources*, **11**, 268–277.

Valière, N., & Berthier, P. (2002). GEMINI: software for testing the effects of genotyping errors and multitubes approach for individual identification. *Molecular Ecology Notes,* **2**, 83–86.

Valière, N. (2002). GIMLET: a computer program for analysing genetic individual identification data. *Molecular Ecology Notes,* **2**, 377–379.

Vilà, C., Amorim, I.R., Leonard, J.A., Posada, D., Castroviejo, J., Petrucci‐Fonseca, F., ... Wayne, R.K. (1999). Mitochondrial DNA phylogeography and population history of the grey wolf Canis lupus. *Molecular Ecology*, **8**, 2089–2103.

**Appendix S2.** **Script for SCR-Poisson Model *M_0_*.** Note that the code to estimate the goodness of fit (GOF) of the model by using the Bayesian p-value approach is also included.

library(nimble)

## define the model

code <- nimbleCode({

psi ~ dunif(0, 1)

alpha0 ~ dnorm(0, 0.1)

alpha2 ~ dnorm(0, 0.1)

sigma ~ dunif(0,5)

log(p0[1:J]) <- alpha0 + alpha2 * E[1:J]

for (i in 1:M) {

z[i] ~ dbern(psi)

s[i, 1] ~ dunif(xlim[1], xlim[2])

s[i, 2] ~ dunif(ylim[1], ylim[2])

d2[i,1:J] <- (s[i,1] - X[1:J,1])^2 + (s[i,2] - X[1:J,2])^2

p[i,1:J] <- z[i] * p0[1:J] * exp(-d2[i,1:J]/(2*sigma^2))

for (j in 1:J) {

outj[i,j] <- sqrt(d2[i,j]) > buffer

y[i,j] ~ dpois(p[i,j])

ynew[i,j] ~ dpois(mu[i,j]*K)

mu[i,j]<-p[i,j]

mu2[i,j]<-mu[i,j]*K

err[i,j] <- pow(pow(y[i,j],.5) - pow(K*mu[i,j],.5),2)

errnew[i,j] <- pow(pow(ynew[i,j],.5) - pow(K*mu[i,j],.5),2)

}

out[i] <- sum(outj[i,1:J])==J

zeros[i] ~ dbern(out[i])

expected[i]<-sum(mu2[i,1:J])

nsum[i]<-sum(y[i,1:J])

nsumnew[i]<-sum(ynew[i,1:J])

err1[i]<- pow(pow(nsum[i],.5) - pow(expected[i],0.5),2)

err1new[i]<- pow(pow(nsumnew[i],.5) - pow(expected[i],0.5),2)

}

for(j in 1:J){

traptotals[j]<- sum(y[1:M,j])

traptotalsnew[j]<-sum(ynew[1:M,j])

expectedtrap[j]<- sum(mu[1:M,j])*K

err3[j]<- pow(pow(traptotals[j],.5) - pow(expectedtrap[j],.5),2)

err3new[j]<- pow(pow(traptotalsnew[j],.5) - pow(expectedtrap[j],.5),2)

}

X1obs<-sum(err[1:M,1:J])

X1new<-sum(errnew[1:M,1:J])

X2obs<-sum(err1[1:M])

X2new<-sum(err1new[1:M])

X3obs<- sum(err3[1:J])

X3new<- sum(err3new[1:J])

N <- sum(z[1:M])

D <- N/area

})

**Appendix S3.** **Simulation studies for SCR estimates under a non-independent Poisson process.**

The spatially explicit capture-recapture approach uses an independent Poisson point process model to estimate the activity centres. This model implies that the individuals are uniformly and independently distributed over the state space (Royle *et al.* 2014). These assumptions are violated by multiple species, including wolves. In order to explore the influence of such violations of SCR assumptions on population size estimates, we carried out three simulation studies. We simulated three datasets: i) a dataset with independent individuals (no aggregation of animals); ii) a dataset with aggregation of individuals in clusters and territorial behaviour randomly distributed within a trap grid and a buffer of$2.5\times\sigma$.; and iii) a dataset with aggregation of individuals in clusters and territorial behaviour randomly distributed within a trap grid.

In the first case (i: simulation independent, Table 1), we simulated an independent dataset with no clusters among individuals. That is, the location of wolves was independent of each other (no packs). In the second case (ii: simulation Strauss inside grid + 2.5*sigma buffer, Table 1), we simulated a dataset with different numbers of clusters of individuals (i.e., packs), starting from a minimum number of 5 packs up to 25 packs (the maximum number of packs to be simulated was established considering the average home range size of subadult/adult wolves in NW Iberia, 122.1±93.6 km^2^; Llaneza 2016, and the size of the study area, ca. 4,378 km^2^; using the same buffer area for a pack territory as in Figure 1 of the main text: 4,378/215 = 20.3 potential packs), data were simulated every 5 packs. Thus, within this dataset we had $N\in\left\{ 5, 10,15, 20,25 \right\}$. Territorial behaviour was also simulated in this dataset. To do this, we simulated repulsion between the centres of clusters using a Strauss distribution with a parameter $a=1$ of repulsion between clusters from the *“spatial”* package in R (Venables & Ripley 2002) under the condition that locations of clusters was randomised in the trap grid and a buffer of$2.5\times\sigma$. Finally, in the third case (iii: simulation Strauss, inside the grid, Table 1), we simulated a dataset following the same procedure every 5 packs as in (ii), but the random location of packs, territorial behaviour and repulsion were simulated only within the trap grid. Simulation repulsion between packs was biologically set to 10 km, taking into account previous known distances between *rendezvous sites* (Jiménez *et al.* 2016). Other parameters to run the simulations were set as follow: For cluster size, we used the average number of wolves per pack in late summer/fall in the study area (Llaneza *et al.* 2012)$(clust.size=8)$; therefore, simulations were based on 40, 80, 120, 160, and 200 individuals; on the other hand, sigma and alpha were set taking into account the results from the present study and Jiménez et al. (2016) ($\sigma=033)$ and alpha ($\alpha_{0}=-0.35)$. Using these scenarios, we tested the influence of the existence of aggregation of individuals in the studied population and the randomness in pack locations across the state space. We simulated the scenarios in R and Nimble (De Valpine *et al.* 2016), each with the same 100 simulations of point/cluster distributions. We ran one chain of the MCMC sampler with 5,000 iterations and 1,000 burn-in.

We computed a frequentist evaluation, using the Root-Mean-Squared-Error (RMSE) for the posterior mean, and the coverage of 95% Highest Posterior Density (HPD) intervals. RMSE was calculated as follows:

$$RMSE=\sqrt{\frac{1}{S}\sum_{1}^{S} (\hat{N}_{(s)}-N)^{2}}$$

, where $\hat{N}$ is the posterior mean for the population size parameter, and N is the number of individuals simulated. Outputs from simulations are shown in Table 1 and Figure 1.

**Table 1.** Results from simulations showing the mean and RMSE of the posterior mean for the population size parameter,$\hat{N}$, and the proportion of the 95% highest posterior density (HPD) intervals covering the data generating value. $\alpha_{0} =-0.35$ and $\sigma=0.33$ for all scenarios.

| Simulation | a | N | Clusters (packs) | $\hat{N}$ | RMSE | Coverage 95%HPD |
| --- | --- | --- | --- | --- | --- | --- |
| i) Independent | - | 40 | - | 42.48 | 6.32 | 92 |
|  |  | 80 |  | 81.57 | 6.93 | 96 |
|  | - | 120 | - | 120.42 | 8.46 | 97 |
|  | - | 160 | - | 161.69 | 9.04 | 97 |
|  |  | 200 |  | 202.09 | 11.30 | 94 |
| ii) Strauss (inside | 1 | 40 | 5 | 40.63 | 8.45 | 75 |
| grid+2.5*sigma buffer) | 1 | 80 | 10 | 81.52 | 10.03 | 83 |
|  | 1 | 120 | 15 | 119.66 | 12.79 | 81 |
|  | 1 | 160 | 20 | 156.23 | 13.35 | 87 |
|  | 1 | 200 | 25 | 191.70 | 15.44 | 84 |
| iii) Strauss (inside | 1 | 40 | 5 | 41.04 | 4.31 | 94 |
| grid) | 1 | 80 | 10 | 81.03 | 5.29 | 97 |
|  | 1 | 120 | 15 | 120.46 | 6.41 | 96 |
|  | 1 | 160 | 20 | 159.84 | 7.19 | 95 |
|  | 1 | 200 | 25 | 198.03 | 8.41 | 94 |

**Figure 1.** Boxplot for population size estimates using different number of random clusters (packs) had $N\in\left\{ 5, 10,15, 20,25 \right\}$. Results from simulations ii (simulation Strauss inside grid + 2.5*sigma buffer, Table 1) are shown in white; whereas results from simulations iii (simulation Strauss, inside the grid, Table 1) are shown in grey. Dotted horizontal red lines denote the total population size simulated (N).


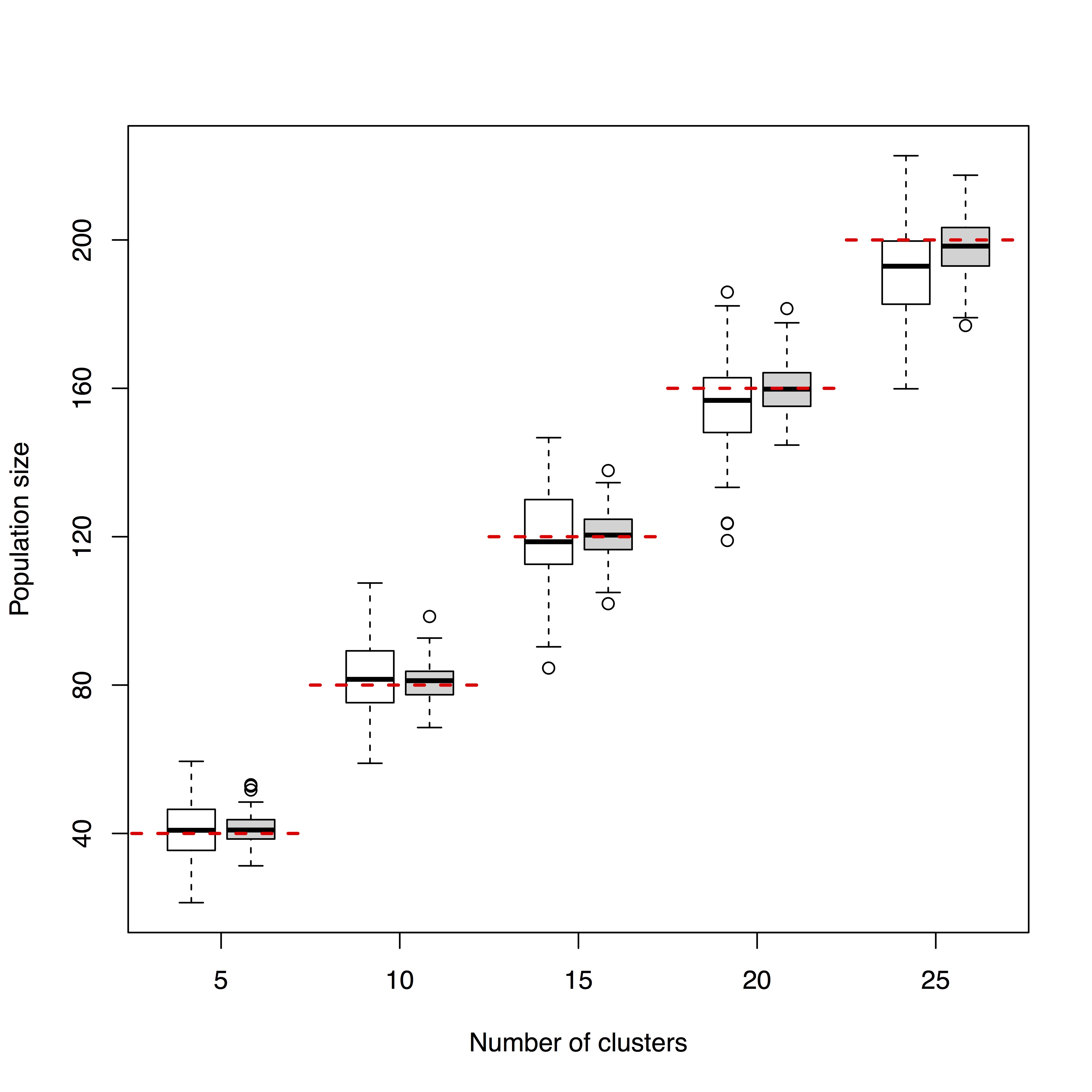


For the simulations considering clusters of individuals and territorial behaviour, we found a slight underestimation in population size estimates (Table 1, Figure 1), similar to that previously reported by Royle (2014) for clusters and by Reich & Gardner (2014) for territorial species. The smaller RMSE was found when all clusters were simulated inside the trap grid. Similarly, the 95% HPD coverage was better if all simulated clusters were inside the trap grid (Table 1, Figure 1). Outside the trap grid, individuals from those randomised distributed clusters were less frequently detected, resulting in a decrease in accuracy and precision in $\hat{N}$ and $\sigma$ (Table 1, Figure 1). In our study case, we did not have clusters outside the trap grid and in the buffer$2.5\times\sigma$. There was a coastline along 65% of the study area, such that reproductive wolf packs have not been traditionally located in the Eastern area (Llaneza et al. 2012b, Llaneza et al. 2015). Therefore, considering the results from simulations (Table 1, Figure 1), the spatial distribution of known reproductive wolf packs within the study area (the trap grid) (Llaneza et al. 2015), and the number of reproductive packs (clusters; n = 11; Llaneza *et al.* 2015), we believe that the aggrupation of wolves in packs and their territorial behaviour had a minimum impact on our population size estimates.

**References**

De Valpine, P., Turek, D., Paciorek, C.J., Anderson-Bergman, C., Lang, D.T., & Bodik, R. (2016). Programming with models: writing statistical algorithms for general model structures with NIMBLE. *Journal of Computational and Graphical Statistics*, 1–28.

Jiménez, J., García, E.J., Llaneza, L., Palacios, V., González, L.M., García‐Domínguez, F., Muñoz-Igualada, J., & López‐Bao, J.V. (2016). Multimethod, multistate Bayesian hierarchical modeling approach for use in regional monitoring of wolves. *Conservation Biology,* **30**, 883-893.

Llaneza, L., García, E.J., Palacios, V., Sazatornil, V., & López-Bao, J.V. (2012). Pack size in the Iberian wolf (*Canis lupus signatus*). Oral communication. Abstract-book of the III Iberian Wolf Congress: p. 19.

Llaneza, L., López‐Bao, J.V., & Sazatornil, V. (2012b). Insights into wolf presence in human‐dominated landscapes: the relative role of food availability, humans and landscape attributes. *Diversity and Distributions,* **18**, 459-469.

Llaneza, L., García, E.J., Palacios, V., & López-Bao, J.V. (2015). Wolf monitoring in Galicia, 2013–2014. Report to the Spanish Ministry of Agriculture, Food and Environment, Spain.

Llaneza, L. (2016). Wolves in human-dominated landscapes of Northwestern Iberian Peninsula. PhD thesis. University of Santiago de Compostela, Spain.

R Core Team (2017). R: A language and environment for statistical computing. R Foundation for Statistical Computing, Vienna, Austria. https://www.R-project.org/.

Reich, B., & Gardner, B. (2014). A spatial capture-recapture model for territorial species. Environmetrics, **25**, 630-637.

Royle, J.A., Chandler, R.B., Sollman, R., & Gardner, B. (2014). Spatial capture-recapture. Elsevier. Academic Press 2014.

Royle, A.J. (2014). How important is the independence-of-s assumption in SCR models? [Online forum comment]. Message posted to

<https://groups.google.com/forum/#!topic/spatialcapturerecapture/>

Venables, W.N., & Ripley, B.D. (2002). Modern Applied Statistics with S. Fourth Edition. Springer, New York.

1) Data simulation Poisson, 1 sampling occasion and independence. Modified from Royle *et al.* (2014)

library(scrbook)

load(“DistIndep.RData”)

simSCR0Poiss<-function (discard0 = TRUE, N=N, K=1, alpha0 = -0.35,

sigma = 0.33, buffer=buffer,

array3d = FALSE, rnd = 2013)

{

set.seed(rnd)

traplocs <- X

Dmat <- e2dist(traplocs, traplocs)

ntraps <- nrow(traplocs)

plot(traplocs)

buffer <- .8

Xl <- min(traplocs[, 1] - buffer)

Xu <- max(traplocs[, 1] + buffer)

Yl <- min(traplocs[, 2] - buffer)

Yu <- max(traplocs[, 2] + buffer)

sx <- runif(N, Xl, Xu)

sy <- runif(N, Yl, Yu)

S <- cbind(sx, sy)

plot(traplocs, pch=3, col='blue', xlim=c(Xl, Xu), ylim=c(Yl, Yu))

points(S, pch=16, col='red')

D <- e2dist(S, traplocs)

alpha1 <- 1/(2 * sigma * sigma)

muy <- exp(alpha0) * exp(-alpha1 * D * D)

Y <- matrix(NA, nrow = N, ncol = ntraps)

for (i in 1:nrow(Y)) {

Y[i, ] <- rpois(ntraps, K * muy[i, ])

}

if (discard0) {

totalcaps <- apply(Y, 1, sum)

Y <- Y[totalcaps > 0, ]

}

dimnames(Y) <- list(1:nrow(Y), paste("trap", 1:ncol(Y), sep = ""))

if (array3d) {

Y <- array(NA, dim = c(N, K, ntraps))

for (i in 1:nrow(Y)) {

for (j in 1:ntraps) {

Y[i, 1:K, j] <- rpois(K, muy[i, j])

}

}

if (discard0) {

Y2d <- apply(Y, c(1, 3), sum)

ncaps <- apply(Y2d, 1, sum)

Y <- Y[ncaps > 0, , ]

}

}

list(Y = Y, traplocs = traplocs, xlim = c(Xl, Xu), ylim = c(Yl,Yu),

N = N, alpha0 = alpha0, sigma = sigma, S=S, K = K, buffer=buffer)

}

## Data simulation

N <- 40 # Population size N=40,80,120,160,200

K <- 1 # sampling occasions

data <- simSCR0Poiss(N=N, K=K, alpha0 = -0.35,

sigma = 0.33, array3d = TRUE, discard0=TRUE)

2) Data simulation Poisson, 1 sampling occasion, with N clusters (=N) and size of cluster (=clust.size); population size (=N* clust.size), and repulsion (>10 km) between clusters. Modified from Royle et al. (2014)

library(scrbook)

load(“DistStrauss.RData”)

simSCR0PoisClustStrauss<-function (discard0 = TRUE, N=N, K=1, alpha0 = -2.5,

sigma = 0.33, clust.size=clust.size,

array3d = FALSE, rnd = 2013)

{

set.seed(rnd)

traplocs <- X

Dmat <- e2dist(traplocs, traplocs)

ntraps <- nrow(traplocs)

plot(traplocs)

buffer <- .8

Xl <- min(traplocs[, 1] - buffer)

Xu <- max(traplocs[, 1] + buffer)

Yl <- min(traplocs[, 2] - buffer)

Yu <- max(traplocs[, 2] + buffer)

library(spatial)

xx <- runif(N, Xl, Xu)

yy <- runif(N, Yl, Yu)

ppregion(Xl, Xu, Yl, Yu)

inds<-cbind(xx,yy)

n <- nrow(inds)

random <- Psim(n)

hardcore <- Strauss(n,r=1)

sxa<-hardcore$x

sya<-hardcore$y

sx<-rep(sxa,clust.size)

sy<-rep(sya,clust.size)

N<- N*clust.size

S <- cbind(sx, sy)

plot(traplocs, pch=3, col='blue', xlim=c(Xl, Xu), ylim=c(Yl, Yu))

points(S, pch=16, col='red')

D <- e2dist(S, traplocs)

alpha1 <- 1/(2 * sigma * sigma)

muy <- exp(alpha0) * exp(-alpha1 * D * D)

Y <- matrix(NA, nrow = N, ncol = ntraps)

for (i in 1:nrow(Y)) {

Y[i, ] <- rpois(ntraps, K * muy[i, ])

}

if (discard0) {

totalcaps <- apply(Y, 1, sum)

Y <- Y[totalcaps > 0, ]

}

dimnames(Y) <- list(1:nrow(Y), paste("trap", 1:ncol(Y), sep = ""))

if (array3d) {

Y <- array(NA, dim = c(N, K, ntraps))

for (i in 1:nrow(Y)) {

for (j in 1:ntraps) {

Y[i, 1:K, j] <- rpois(K, muy[i, j])

}

}

if (discard0) {

Y2d <- apply(Y, c(1, 3), sum)

ncaps <- apply(Y2d, 1, sum)

Y <- Y[ncaps > 0, , ]

}

}

list(Y = Y, traplocs = traplocs, xlim = c(Xl, Xu), ylim = c(Yl,Yu),

N = N, alpha0 = alpha0, sigma = sigma, S=S, K = K, buffer=buffer)

}

## Data simulation

N <- 5 # Number of clusters (5,10,15,20,25)

clust.size<-8 # cluster size

NT<- N*clust.size; NT # Population size

K <- 1 # sampling occasions

data <- simSCR0PoisClustStrauss (N=N, clust.size=clust.size, K=K,

alpha0 = -0.35, sigma = 0.33, array3d = TRUE, discard0=TRUE)

***Data Availability Statement:**

R Data archives “DistStrauss.RData” and “DistIndep.RData” are available upon request. Contact José Vicente López-Bao ([jv.lopezbao@gmail.com)](mailto:jv.lopezbao@gmail.com)) or José Jiménez ([Jose.Jimenez@uclm.es)](mailto:Jose.Jimenez@uclm.es)).
